# Comparing content within a culturally-adapted digital treatment for Hispanic patients with alcohol use disorder

**DOI:** 10.1038/s41746-025-02197-7

**Published:** 2025-12-08

**Authors:** Bryan Benitez, Tami Frankforter, Charla Nich, Manuel Paris, Brian D. Kiluk

**Affiliations:** Yale University, School of Medicine, New Haven, CT USA

**Keywords:** Human behaviour, Phase II trials

## Abstract

Hispanic people in the U.S. experience alcohol-related harms comparable to non-Hispanic White people. Hispanic people often face more challenges to accessing alcohol use disorder (AUD) treatment. Culturally-adapted digital therapeutics may improve treatment access. Using data from two clinical trials of a culturally-adapted digital cognitive behavioral therapy program for Spanish-speaking Hispanic people (CBT4CBT-S, *n* = 43), we identified which program topics were most strongly linked to outcomes (NCT03474588 registered on 3/15/2018 and NCT02043210 registered on 1/10/2014 at clinicaltrials.gov). Topics were not administered in a randomized order. Participants drank less alcohol after completing content about coping with craving (*OR* = 0.48), cognitive restructuring (*OR* = 0.73), and problem solving (*OR* = 0.79). Participants were slightly more favorable towards content about cognitive restructuring (*b* = 0.14) and decision making (*b* = 0.09). These findings can inform further development of culturally-adapted digital therapeutics for AUD, which may improve alcohol-related health outcomes for Hispanic people.

## Introduction

Alcohol use disorder (AUD) is often characterized by strong expectations of reward from alcohol use, patterns of excessive alcohol use, and amplified negative emotionality when discontinuing alcohol use^1,2^. About 10% of the U.S. population meets criteria for AUD, with more than 140,000 alcohol-related deaths per year^3–5^. Although Hispanic people are affected by AUD at rates comparable to non-Hispanic White people, they are at increased risk for alcohol-associated liver disease^4,6,7^. Less than 10% of Hispanic people with AUD have received any treatment for alcohol use in the past year, partly because of linguistic differences, socioeconomic status, ethnic discrimination, and barriers to culturally-adapted treatments^8–12^. Shortages of trained treatment providers also limit access to treatment for AUD and other substance use disorders (SUDs) in traditional clinic settings^13,14^.

Digital therapeutics (i.e., software that deliver treatments for medical conditions or diseases) have significant potential to make evidence-based treatment for AUD more accessible, especially among disadvantaged groups^15–20^. However, few studies of digital therapeutics for AUD and SUDs have been conducted primarily with Hispanic people^21^. Computer-Based Training for Cognitive Behavioral Therapy (CBT4CBT) is a digital therapeutic that teaches CBT skills for improving executive control over prepotent behaviors and decreasing motivation to use alcohol/substances^16^. Multiple clinical trials have supported its efficacy at reducing rates of alcohol and substance use^22–27^. A culturally-adapted version of CBT4CBT for Spanish-speaking Hispanic people has also been developed (“CBT4CBT-S”)^27–29^. Like the English versions of CBT4CBT, CBT4CBT-S contains distinct modules that teach skills through narrator-guided didactic content, interactive practice exercises, and modeling of skills through video vignettes with professional actors. The cultural adaptation of CBT4CBT-S also demonstrates CBT skills through dramatized portrayals of an immigrant family whose members experience stressors related to AUD and SUDs^30^. Efficacy of CBT4CBT-S as an adjunct to standard outpatient care for AUD and SUDs has been supported in a previous randomized clinical trial and is being evaluated in a second recently completed trial^27,31^. Importantly, the modularized format of CBT4CBT facilitates identifying which program topics are most useful for reducing alcohol/substance use^16^. Comparing content within a culturally-adapted digital therapeutic, like CBT4CBT-S, may distinguish what mechanisms of AUD treatment are most potent among Hispanic patients, who are underrepresented in clinical trials of AUD and SUDs^16,32,33^.

To advance the development of digital therapeutics for Hispanic people with AUD, the current study sought to identify which CBT4CBT-S modules were associated with the least amount of alcohol use and regarded most favorably by patients. Furthermore, associations between alcohol use and specific modules may vary depending on the daily risk of drinking. Socially-significant days, such as weekends (Fridays-Sundays), holidays, and birthdays, are strong predictors of increased risk for alcohol use, craving, and heavy drinking in many populations^34–38^. When there is greater risk of an infrequent negative outcome, precision medicine research suggests that larger treatment effects may be observed^39–41^. Therefore, we hypothesized that differences in drinking rates associated with CBT4CBT-S modules would be larger on days of greater risk for drinking.

The current study included data from two randomized clinical trials that administered CBT4CBT-S in addition to standard outpatient treatment for AUD/SUDs^31,42^. In both trials, CBT4CBT-S taught the same core CBT skills, but differed in the type of substance use being targeted. In the first trial, the CBT4CBT-S content broadly covered several substances (including alcohol), and patients with AUD and/or SUDs were enrolled^27,28,42^. In the second trial, the CBT4CBT-S content targeted alcohol use more specifically, and only patients with AUD were enrolled^31^. For the current study, we evaluated outcomes of patients who had an AUD diagnosis. Except where noted in the “Methods” section, the two clinical trials followed practically identical study procedures.

## Results

Data from the first trial was collected between March 2014 and December 2016. Data from the second trial was collected between August 2019 and February 2023. For the current study, a total of 47 participants met the eligibility criteria across both clinical trials. To be included, participants must have completed at least one CBT4CBT-S module and one observation of the outcome (*n* = 43 for alcohol use; *n* = 42 for favorability ratings). There were 20 participants from the first trial (47% of those randomized to CBT4CBT-S) and 23 from the second trial (100% of those randomized to CBT4CBT-S). All participants spoke Spanish as their preferred language and identified as Hispanic (40% Puerto Rican, 21% Mexican, 21% other Central American countries, 19% other countries). Most were male (79%) and over 40 years old (*M* = 42.6, *Mdn* = 42, *SD* = 11.4). A few had another SUD diagnosis besides AUD (28%). The ASI alcohol composite scores were very similar between participants diagnosed with only AUD (*M* = 0.36, *SD* = 0.19) and those diagnosed with both AUD and another SUD (*M* = 0.34, *SD* = .20). The ASI alcohol composite scores were comparable to those of other clinical populations (*M* = 0.35, *SD* = 0.19)^43,44^. No serious adverse events were attributed to CBT4CBT-S.

Table 1 shows descriptive statistics for variables created from the alcohol calendar data. Table 2 shows descriptive statistics for patient feedback form items. Most participants completed all 6 modules (63%), and a substantial majority completed at least 4 modules (84%). Most modules (83%) were completed on low-risk drinking days. A total of 1219 non-missing daily data from the alcohol calendars were analyzed for the alcohol use outcome (524 missing observations). A total of 1192 non-missing ratings of individual items from 199 completed patient feedback forms were analyzed for the patient favorability outcome (320 missing ratings). Only one participant was missing the ASI alcohol composite score.

**Table 1:** Descriptive statistics for alcohol calendar variables by daily risk of alcohol use and CBT4CBT-S modules

Risk of alcohol use	Module	Days *N*	Participants *N*	Alcohol use *M* (*SD*)	Days since treatment randomization *M* (*SD*)	Alcohol use in previous week *%*
Low-risk day	Functional analysis	147	41	0.09 (0.20)	10.51 (8.49)	54%
	Assertive communication	104	30	0.11 (0.25)	28.27 (15.85)	47%
	Coping with craving	105	28	0.02 (0.07)	27.71 (10.08)	50%
	Cognitive restructuring	108	30	0.05 (0.19)	25.53 (11.78)	43%
	Problem solving	91	27	0.08 (0.19)	38.48 (7.89)	41%
	Decision making	114	31	0.10 (0.24)	33.16 (14.34)	35%
High-risk day	Functional analysis	117	39	0.22 (0.29)	12.41 (8.71)	54%
	Assertive communication	100	30	0.22 (0.33)	29.70 (15.21)	47%
	Coping with craving	82	27	0.11 (0.20)	30.00 (10.13)	48%
	Cognitive restructuring	84	27	0.15 (0.25)	26.81 (12.97)	44%
	Problem solving	76	25	0.19 (0.31)	39.72 (7.62)	44%
	Decision making	91	30	0.22 (0.31)	35.03 (14.38)	37%

*Note*. Generated from calendars of daily alcohol use reported by participants. Low-risk day = any day that was not a high-risk drinking day. High-risk day = Friday, Saturday, Sunday, or holiday (and the day before the holiday) associated with increased drinking. Days *N* = number of non-missing daily calendar observations. Participants *N* = number of participants with at least 1 non-missing daily calendar observation. Alcohol use = proportions of days on which alcohol use was reported. *M* = mean. *SD* = standard deviation. % = percentage of calendar observations wherein alcohol use was reported at least once in the previous week.

**Table 2:** Descriptive statistics for patient favorability ratings by CBT4CBT-S modules

Module	*N*	Effectiveness *M* (*SD*)	Novelty *M* (*SD*)	Applicability *M* (*SD*)	Navigation *M* (*SD*)	Enjoyability *M* (*SD*)	Relatability *M* (*SD*)
Functional analysis	39	2.74 (0.88)	2.21 (1.17)	2.31 (1.10)	2.69 (1.08)	2.51 (1.05)	2.18 (1.12)
Assertive communication	34	2.76 (0.92)	2.26 (1.26)	2.53 (1.08)	2.76 (0.96)	2.65 (1.04)	1.97 (1.19)^a^
Coping with craving	31	2.74 (1.06)	2.23 (1.18)	2.39 (1.26)	2.81 (1.05)	2.45 (1.12)	2.23 (1.28)
Cognitive restructuring	36	3.08 (0.77)	2.25 (1.11)	2.56 (1.11)	2.89 (0.85)	2.58 (1.11)	2.33 (1.01)
Problem solving	31	2.74 (1.12)	2.16 (1.19)	2.35 (1.23)	2.61 (0.99)	2.48 (1.23)	2.23 (1.25)^b^
Decision making	28	2.71 (1.12)	2.36 (1.06)	2.25 (1.29)	2.57 (1.26)	2.57 (1.20)	2.18 (1.33)

*Note*. Generated from ratings of individual items on the patient feedback form. The Likert scales for the ratings ranged from 0 (“not at all”) to 4 (“a lot”).

*N* number of non-missing ratings, *M* mean, *SD* standard deviation.

^a^The number of non-missing ratings for these statistics is 33.

^b^The number of non-missing ratings for these statistics is 30.

### Alcohol use

**Fig. 1:**
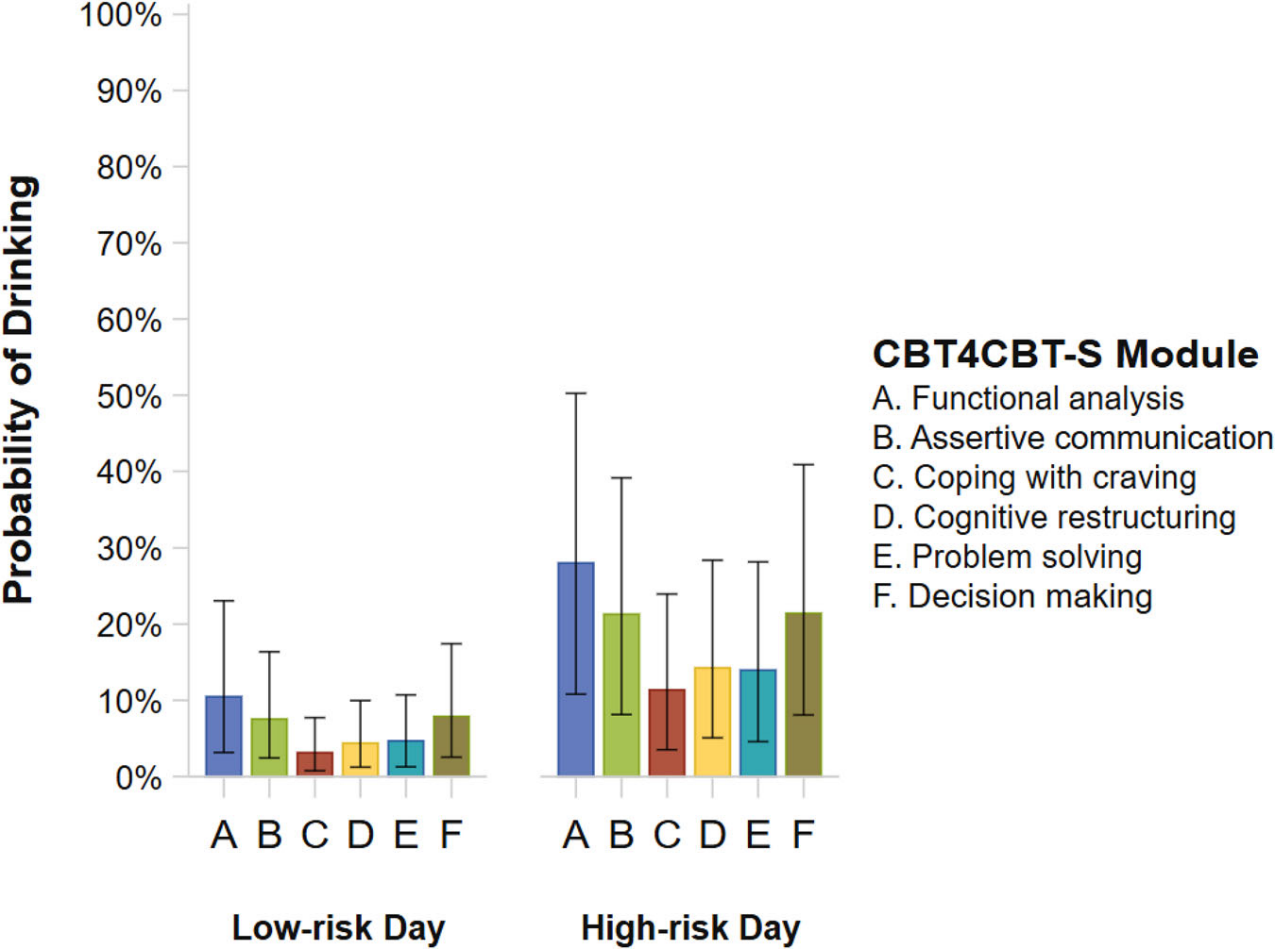
Predicted probabilities based on marginal means generated from the Bayesian MMRM logistic regression model of daily alcohol use. Error bars represent 95% credibility intervals. Low-risk day = any day that was not a high-risk drinking day. High-risk day = Friday, Saturday, Sunday, or holiday (and the day before the holiday) associated with increased drinking. Figures were generated using SAS 9.4.

High-risk drinking days were associated with over a 200% increase in the odds of alcohol use (*OR* = 3.83, 95% CI[1.96, 6.90]). Similarly, self-reported alcohol use in the previous week was associated with over a 200% increase in the odds of alcohol use during the current week (*OR* = 3.17, 95% CI[1.51, 5.84]). On both low- and high-risk drinking days, the CBT4CBT-S modules associated with the least amount of alcohol use were coping with craving, cognitive restructuring, and problem solving (Table 3; Fig. 1). The coping with craving module had the highest posterior probability (> 95%) of decreased drinking when compared to the other modules, with an expected reduction in the odds of drinking around 50%. The cognitive restructuring and problem solving modules had the next highest posterior probabilities (>=80%) of decreased drinking when compared to other modules, with an expected reduction in the odds of drinking around 20%. Our hypothesis that the differences in drinking between modules would be greater on high-risk drinking days was not supported. The direction and magnitude of the differences were very similar on low- and high-risk drinking days (Table 3).

**Table 3:** Differences in alcohol use between CBT4CBT-S modules by daily risk of drinking

Daily risk of drinking	Module	*OR* [*95 CI*]	*p*(*H*_*1*_*|data*)
Low-risk day	Functional analysis	2.34 [1.03, 4.79]	2%
	Assertive communication	1.49 [0.73, 2.67]	14%
	Coping with craving	0.48 [0.16, 0.95]	98%
	Cognitive restructuring	0.73 [0.31, 1.37]	86%
	Problem solving	0.79 [0.35, 1.46]	80%
	Decision making	1.57 [0.79, 2.91]	12%
High-risk day	Functional analysis	2.24 [1.08, 4.35]	2%
	Assertive communication	1.40 [0.75, 2.36]	15%
	Coping with craving	0.56 [0.24, 1.05]	97%
	Cognitive restructuring	0.77 [0.37, 1.36]	84%
	Problem solving	0.74 [0.35, 1.33]	86%
	Decision making	1.41 [0.73, 2.42]	16%

*Note*. Results for the Bayesian MMRM logistic regression model of daily alcohol use. Low-risk day = any day that was not a high-risk drinking day. High-risk day = Friday, Saturday, Sunday, or holiday (and the day before the holiday) associated with increased drinking. Odds ratios and posterior probabilities were based on contrasts between the marginal mean for each module ($\hat{{\boldsymbol{\mu }}}$_***a***_) and the average of the marginal means for the other modules ($\hat{{\boldsymbol{\mu }}}$_***g***_). *OR* = odds ratio. *95 CI* = 95% credibility interval. *p*(*H*_*1*_*|data*) = posterior probability that the module is associated with less drinking than the other modules (*H*_*1*_:$\hat{{\boldsymbol{\mu }}}$_***a***_ < $\hat{{\boldsymbol{\mu }}}$_***g***_).

### Patient favorability

The CBT4CBT-S modules rated most favorably by participants were cognitive restructuring and decision making (Table 4). Those two modules consistently had the highest posterior probabilities (> 80%) of more favorable ratings when compared to the other modules. Across the various feedback form items, the expected mean differences between the cognitive restructuring module and the other modules ranged from 0.10 to 0.14. The expected mean differences between the decision-making module and the other modules ranged from 0.08 to 0.11. These mean differences between modules were relatively small, given that the standard deviations of the feedback ratings were around 1 (Table 2).

**Table 4:** Differences in patient favorability ratings between CBT4CBT-S modules by feedback item

Item	Module	*b* [*95 CI*]	*p*(*H*_*1*_*|data*)
Effectiveness	Functional analysis	−0.07 [−0.25, 0.10]	22%
	Assertive communication	−0.06 [−0.22, 0.10]	24%
	Coping with craving	−0.05 [−0.22, 0.11]	29%
	Cognitive restructuring	0.14 [−0.02, 0.32]	96%
	Problem solving	−0.06 [−0.24, 0.11]	24%
	Decision making	0.09 [−0.08, 0.27]	85%
Novelty	Functional analysis	−0.07 [−0.25, 0.11]	23%
	Assertive communication	−0.05 [−0.21, 0.11]	27%
	Coping with craving	−0.04 [−0.20, 0.12]	32%
	Cognitive restructuring	0.10 [−0.06, 0.26]	89%
	Problem solving	−0.06 [−0.24, 0.10]	23%
	Decision making	0.11 [−0.06, 0.30]	90%
Applicability	Functional analysis	−0.07 [−0.26, 0.09]	20%
	Assertive communication	−0.03 [−0.19, 0.14]	33%
	Coping with craving	−0.04 [−0.21, 0.11]	31%
	Cognitive restructuring	0.12 [−0.04, 0.30]	92%
	Problem solving	−0.06 [−0.24, 0.11]	25%
	Decision making	0.08 [−0.09, 0.26]	83%
Navigation	Functional analysis	−0.06 [−0.24, 0.11]	23%
	Assertive communication	−0.05 [−0.21, 0.12]	28%
	Coping with craving	−0.03 [−0.18, 0.13]	37%
	Cognitive restructuring	0.13 [−0.04, 0.30]	94%
	Problem solving	−0.07 [−0.25, 0.09]	21%
	Decision making	0.08 [−0.09, 0.26]	83%
Enjoyability	Functional analysis	−0.06 [−0.24, 0.11]	23%
	Assertive communication	−0.04 [−0.20, 0.13]	31%
	Coping with craving	−0.05 [−0.22, 0.11]	28%
	Cognitive restructuring	0.11 [−0.06, 0.28]	91%
	Problem solving	−0.06 [−0.24, 0.10]	24%
	Decision making	0.10 [−0.07, 0.29]	88%
Relatability	Functional analysis	−0.06 [−0.24, 0.11]	24%
	Assertive communication	−0.08 [−0.26, 0.08]	18%
	Coping with craving	−0.03 [−0.20, 0.12]	34%
	Cognitive restructuring	0.12 [−0.03, 0.29]	93%
	Problem solving	−0.05 [−0.23, 0.12]	28%
	Decision making	0.10 [−0.08, 0.29]	87%

*Note*. Results for the Bayesian MMRM linear regression model of patient feedback form items. Differences and posterior probabilities were based on contrasts between the marginal mean for each module ($\hat{{\boldsymbol{\mu }}}$_***a***_) and the average of the marginal means for the other modules ($\hat{{\boldsymbol{\mu }}}$_***g***_). *b* = unstandardized mean difference. *95% CI* = 95% credibility interval. *p*(*H*_*1*_*|data*) = posterior probability of the hypothesis that the module was rated better than the other modules (*H*_*1*_:$\hat{{\boldsymbol{\mu }}}$_***a***_ > $\hat{{\boldsymbol{\mu}}}$_***g***_).

### Sensitivity analyses

Results of the sensitivity analyses that only included data from participants who completed all 6 core modules were similar to the findings with the full sample (Supplementary Tables 5−6). Coping with craving, cognitive restructuring, and problem solving modules were still associated with the greatest reductions in alcohol use. However, the magnitude of the contrast between the coping with craving module and the other modules was not as pronounced. Our hypothesis that the contrasts between modules would be greater on high-risk drinking days was still not supported. Cognitive restructuring and decision making modules were again rated most favorably by participants across all items.

## Discussion

The findings provide evidence for which topics from a culturally-adapted digital CBT program were most helpful for Hispanic people with AUD. Alcohol consumption was least likely after participants engaged with CBT4CBT-S content about coping with craving, problem solving, and cognitive restructuring. The coping with craving module was notable for being associated with about 50% less odds of drinking when compared to the other modules collectively. However, participants did not favor the coping with craving module more than the other modules. Instead, participants were slightly more favorable towards the cognitive restructuring and decision-making modules. Therefore, although content about coping with craving had the strongest association with less drinking, content about cognitive restructuring was associated with both less drinking and better patient favorability.

Contrary to our hypothesis, the daily risk of drinking did not moderate the association between alcohol use and the CBT4CBT-S content. The absence of a moderation effect may be partially explained by the fact that less than 20% of modules were completed on high-risk drinking days (i.e., Fridays-Sundays, holidays). Previous studies of outpatient treatment for AUD/SUDs found greater reductions in motivation to use alcohol/substances on the days when treatment sessions were completed and shortly thereafter^45,46^. Thus, the current study may have had insufficient numbers of modules completed on high-risk drinking days to robustly test our moderation hypothesis. We did, however, find that weekends (Fridays-Sundays) and holidays were associated with a substantial increase in the odds of drinking (> 200%), which suggests weekends and holidays are important proxies for periodic factors that motivate drinking among Hispanic people with AUD.

Our findings can also inform the design of condensed and targeted interventions that are administered rapidly and remotely through mobile devices, which is a nascent area of research with the Spanish-speaking Hispanic population^47^. Mobile delivery of condensed digital therapeutics can be especially valuable for targeting periods of high risk for drinking throughout the week or within a day^15^. However, a recent review concluded that the overall evidence for the effectiveness of current mobile app interventions for AUD is mixed and of moderate quality at best^48^. Engagement with mobile technology is often brief and sporadic, and so content delivered through mobile devices needs to maximize treatment efficacy within a short period. The clinical trials from which our study data originated did not measure alcohol use throughout the day, but the observed differences in drinking between CBT4CBT-S modules occurred within a relatively short period (i.e., 6 days or less after completing each module). In this context, content about coping with craving may have been associated with less drinking because the prescribed behaviors can be implemented rapidly without much planning or practice (e.g., distraction; relaxation). Our findings are consistent with reviews suggesting that coping skills are among the most robust mediators of treatment outcomes for AUD and SUDs^32,49,50^. In a prior study, we also found that CBT4CBT-S improved participants’ coping skills and that these improvements were associated with future reductions in substance use^29^. Therefore, digital content providing skills training related to coping with craving may have a relatively strong and immediate influence on motivation for drinking, which is valuable for mobile treatment delivery during high-risk periods for drinking.

Overall, participants had favorable impressions of each CBT4CBT-S module, consistent with previous findings that most patients were satisfied with using CBT4CBT^22,25,30^. The similarity in positive responses to each module may be because all the content was conveyed through a common format of didactic teaching interspersed with practice exercises and dramatizations, all of which were culturally adapted for Hispanic people^30^. Previous research supports the utility of culturally adapting treatments for AUD and SUDs^51–53^. Incorporating the patient’s culture into the design of digital therapeutics is also emphasized by principles of social learning, which form the basis of cultural processes^54,55^. CBT4CBT-S was adapted for and tested in a general sample of U.S. Hispanic patients, but just as English speakers are not a homogenous group, some Spanish speakers may respond differently to CBT4CBT-S depending on how alcohol is viewed within their specific culture (e.g., prevalence of binge drinking in country of origin)^56,57^. Therefore, digital therapeutics tailored for specific patient subgroups will almost certainly benefit from incorporating cultural features of the intended patient population.

The most significant limitation to our study is the lack of randomization to the order in which modules were assigned. Therefore, we cannot make strong inferences regarding the causal effects of the digital content on alcohol use or patient favorability. Since all participants were also enrolled in TAU, our results do not reflect the use of digital treatment content independent of standard outpatient treatment for AUD. We did, however, statistically control for recent alcohol use and time since randomization to account for generic changes that are unrelated to specific digital content (e.g., cumulative effects of being in treatment; regression to the mean). The number of participants in our study was modest, limiting both the statistical precision and the generalizability of our findings to the larger Hispanic population. The clinical trials in our study enrolled only Hispanic patients who were fluent in Spanish. Future clinical studies should evaluate what types of digital treatment content for AUD may be most effective in other cultural groups, including Hispanic people who are not fluent in Spanish. Future clinical trials may also need to devote greater effort to recruit Spanish-speaking Hispanic women, who may be underrepresented in AUD trials because they are less likely to report alcohol use than Spanish-speaking men^56^. The relatability ratings of the modules were slightly lower than expected (see Table 2). It is possible that participants, many of whom were unemployed, did not relate to some aspects of CBT4CBT-S because the dramatized vignettes depicted more typical socioeconomic circumstances faced by U.S. Hispanic people (e.g., low-wage employment, stable housing). We are actively investigating patients’ perceptions of CBT4CBT-S content by conducting mixed-methods research with individuals from clinics that enrolled participants for the clinical trials of CBT4CBT-S (e.g., user-centered design focus groups). The first trial was conducted during 2014-2016 and required participants to access CBT4CBT-S at the clinic sites, whereas digital interventions are now commonly deployed remotely on mobile devices and may be accessed on demand. Our study is also unable to distinguish which specific content within each module might be most efficacious (e.g., didactic content, practice exercises, dramatizations). To assess the importance of specific content, future studies would benefit from randomizing the order of modules and dividing the digital content into smaller units. Randomizing the sequence of condensed digital units would be informative for developing mobile versions of digital therapeutics to deliver treatment outside of traditional clinic settings. Ultimately, these types of clinical studies could make evidence-based treatments for AUD more accessible to underserved populations.

Our study identified which CBT skill topics within a culturally-adapted digital therapeutic (CBT4CBT-S) may be most helpful for Hispanic people with AUD. Modules about coping with craving, problem solving, and cognitive restructuring were most strongly linked to decreased drinking. Although weekends and holidays were associated with substantially increased risk for drinking, they did not moderate the associations between modules and alcohol use. When providing feedback about each module, participants slightly favored content about cognitive restructuring and decision making. Future studies should use randomized designs and mobile technology to explore which digital content are most effective outside of traditional clinic settings for AUD. Doing so can contribute to more accessible and effective treatments for Hispanic patients with AUD.

## Methods

### Participants

Both trials were registered in clinicaltrials.gov (NCT03474588 on 3/15/2018 and NCT02043210 on 1/10/2014), were approved by the Yale University Institutional Review Board, and obtained informed consent from all participants. The CONSORT diagram and checklist are reported in Supplementary Fig. 1 and Supplementary Table 7^58^. The eligibility criteria included being primarily Spanish speaking, 18 years of age or older, able to commit to 8 weeks of outpatient treatment, and not having an untreated psychotic or bipolar disorder. For the first trial, participants had to be seeking outpatient treatment for alcohol/substance use and meet criteria for current abuse or dependence of alcohol, cocaine, marijuana, opioid, or other stimulant according to the 4^th^ edition of the American Psychiatric Association’s Diagnostic and Statistical Manual (DSM-IV)^59^. In the second trial, participants had to be seeking outpatient treatment for alcohol use and could not meet criteria for another current SUD (other than nicotine) according to DSM-5 criteria^60^. We only included participants who had a current diagnosis of DSM-5 AUD or DSM-IV alcohol abuse/dependence. Given the similar clinical profiles between the DSM-5 and DSM-IV categories, we refer to both participant groups as having an “AUD” diagnosis^61,62^. Participants were recruited with advertisements at the clinics and/or referred by clinic staff. The clinics were community mental health facilities that primarily served individuals from under-resourced backgrounds (e.g., unemployed, no insurance).

### Interventions

Participants were randomly assigned to receive 8 weeks of either treatment as usual (TAU) or TAU plus access to CBT4CBT-S. TAU consisted of weekly individual and/or group counseling at an outpatient facility that also offered pharmacotherapy and social work services. An urn randomization scheme was designed to balance prognostic baseline variables (e.g., gender, proficiency with computers, preferred drug type). Patients and study staff who administered the treatment and assessments were not blinded to the treatment condition. Since our goal was to compare content within the digital therapeutic, we included only participants assigned to TAU plus CBT4CBT-S. CBT4CBT-S was accessed using a computer or tablet within a private office space located at the treatment site, and a study staff member was available to assist as needed. However, to adjust for societal disruption caused by the COVID-19 pandemic that occurred during the second trial, 18 participants in the second trial were provided with cellular-enabled tablets to access CBT4CBT-S remotely instead of at the clinic. Participants were compensated for completing assessments at baseline, weekly during the treatment period, and monthly during the 6-month follow-up period. Described in detail elsewhere, CBT4CBT-S consists of 7 digital modules that teach CBT principles and skills through culturally-adapted didactics and dramatizations (*telenovela* series) of common situations experienced by Hispanic people with AUD or SUDs^30^. The cultural adaptation process included bicultural and bilingual Hispanic American research and clinical staff discussing ideas and getting feedback for CBT4CBT-S content from Spanish-speaking Hispanic clinicians and patients at a large outpatient mental health clinic. Although Hispanic communities in the U.S. are heterogenous (e.g., different countries of origin, socioeconomic backgrounds), specific cultural values and expressions that are shared among many Hispanic people were explicitly integrated into the CBT4CBT-S content (e.g., *confianza*, *familismo*, *respeto*). Furthermore, certain program features were adapted to ensure accessibility to a wide target audience (i.e., addressing differences in literacy by including audio options for all written content). Each module is self-paced and requires about 35–45 min to complete; participants were asked to complete one module per week. Across all versions of CBT4CBT, including CBT4CBT-S, there are 6 core modules that cover CBT topics as outlined in therapist manuals^63,64^: functional analysis (identifying patterns of substance use), assertive communication (refusing offers to use substances), coping with craving (regulating desires to use substances), cognitive restructuring (changing unhelpful thoughts), problem solving (evaluating problems and potential solutions), and decision making (identifying how any decision can affect the risk of substance use)^16^. The 7th module differed between the two CBT4CBT-S trials; preventing HIV was covered in the first trial, while addressing trauma was covered in the second trial. For the purposes of the current study, we only analyzed data associated with the 6 core modules common to both trials. The order in which participants completed the modules was not randomized. In the first trial, participants were required to complete the modules in the same sequence as listed above. In the second trial, participants could complete each module in whatever order they preferred. Even when the same order of modules was followed, the number of days between randomization and when each module was completed varied between participants because of different weekly schedules and missed visits.

### Measures

#### Alcohol Consumption

The timeline followback calendar method was used to record self-reported daily alcohol use during the 8-week treatment period and subsequent follow-up period^65^. Each day on the calendar was categorized as either a day when any drinking occurred (= 1) or a day of abstinence from alcohol (= 0). To longitudinally structure the data, the date when a participant completed each CBT4CBT-S module was matched to their alcohol calendar data for that day and the 6 days immediately afterwards. The longitudinal data structure of 7 days was chosen to generalize to the weekly pattern of outpatient AUD therapy, wherein sessions typically occur on the same day each week. Furthermore, previous evidence shows that the effects of psychotherapy on motivation for alcohol/substance use are likely to be stronger on days when treatment sessions are completed and shortly thereafter^45,46^. Therefore, we assume that any differences in alcohol use between CBT4CBT-S modules are most likely to occur within a week after completing each module.

To distinguish each module’s unique association with drinking, calendar data associated with each module only included days before the date when the next CBT4CBT-S module was completed. If more than one module was completed on the same date, calendar data associated with those modules were excluded. Missing observations of daily alcohol use were inserted, as needed, to ensure that each module was linked with 7 days for each participant. Each calendar day was categorized as a high-risk drinking day if it was on the weekend (Fridays-Sundays) or on a holiday (and the day before the holiday) that is associated with increased drinking^34–38^. All other days were categorized as low-risk drinking days. Missing observations of daily alcohol use were categorized so that at least 3 of the 7 total days associated with each module were high-risk drinking days, since each calendar week includes at least 3 high-risk drinking days (Fridays-Sundays; Supplementary Tables 1-3).

#### Patient favorability

Participants’ favorability of the CBT4CBT-S modules were measured using a patient feedback form that was administered to participants after they completed each module (available at https://osf.io/encv6/). The form included six separate questions about the content of each module, specifically its effectiveness, novelty, personal applicability, ease of navigation, enjoyability, and personal relevance of the dramatizations. Participants rated their response to each question using a 5-point Likert scale (0 = “not at all”; 4 = “a lot”). The feedback form also included a binary question about homework completion and a binary question about technical errors encountered while using the program. Two optional open-ended questions about technical errors and generic feedback were asked but elicited few responses. Only the Likert scale questions were analyzed in the current study to specifically compare patients’ favorability towards the various types of digital content. Missing observations were inserted, as needed, to ensure that each participant had a potential rating linked to every item and module.

#### AUD severity

The Spanish version of the Addiction Severity Index (ASI) was used as a baseline measure of AUD severity^66^. The ASI assesses the severity of problems across multiple domains relevant to addiction, including medical, psychiatric, social, and alcohol/substance use. The current study utilized the composite scores for the alcohol domain, which could range from 0 to 1^67^.

### Statistical analyses

The planned sample sizes for both clinical trials were based on power analyses that compared TAU and TAU plus CBT4CBT-S in a parallel group design. No interim analyses or stopping rules were used. The exploratory analyses conducted in the current study were not based on a priori power analyses. Although the order in which CBT4CBT-S modules were delivered was not randomized, we statistically controlled for changes in outcomes over time by covarying for the number of days since treatment randomization. This covariate mitigates the influence of potential confounds that are related to non-specific changes over time (e.g., cumulative effects of being in treatment; regression to the mean). Since all participants included in the current study were assigned to complete all CBT4CBT-S modules, comparing outcomes between modules is essentially a within-person comparison, which can alleviate some concerns about confounding related to treatment settings or participant characteristics (e.g., clinic resources; response styles). When analyzing alcohol use as an outcome, we also statistically controlled for alcohol use in the 7 days prior to completing a module to mitigate the influence of autocorrelation in drinking behavior over time. Furthermore, analysis of the alcohol use outcome statistically controlled for whether a specific calendar date was associated with greater risk for drinking (e.g., Fridays, Saturdays, Sundays, holidays), which mitigates the influence of important periodic motivators for drinking.

To compare the associations between alcohol use and specific modules, the unit of analysis was the day recorded on the alcohol timeline follow back calendar. To compare participants’ favorability towards specific modules, the unit of analysis was each item on the patient feedback form. The lack of independence in the units of analysis was accounted for statistically by including a fixed effect for the clinical trial study identifier and random effects for the participant identifier and the patient feedback form item identifier. Hypothesis tests were conducted with posterior samples generated by Bayesian mixed models for repeated measures (MMRM) using Markov chain Monte Carlo procedures (MCMC)^68^. We used Bayesian inference to determine which modules had the greatest posterior probability of being superior on the outcomes. To test if a specific module was associated with the outcome, the marginal mean for that module ($\hat{{\rm{\mu }}}$_*a*_) was contrasted with the average of the marginal means for the other 5 modules ($\hat{{\rm{\mu }}}$_*g*_; Supplementary Table 4). Inferences were made with posterior probabilities, defined as the percentage of posterior MCMC samples where $\hat{{\rm{\mu }}}$_*a*_ < $\hat{{\rm{\mu }}}$_*g*_ for the alcohol use outcome (i.e., less drinking) or $\hat{{\rm{\mu }}}$_*a*_ > $\hat{{\rm{\mu }}}$_*g*_ for the favorability outcome (i.e., better feedback ratings). To estimate effect sizes for the alcohol use outcome, the contrasts were converted to odds ratios with 95% equal-tailed credibility intervals (95% CI). To estimate effect sizes for the favorability outcome, the unstandardized coefficients for the contrasts were interpreted directly as mean differences with 95% CI.

Alcohol use was modelled with a binomial logistic regression. Fixed effect predictors of alcohol use were age, sex, ASI alcohol composite score, clinical trial identifier, number of days since treatment randomization, indicator of a high-risk drinking day, and indicator of alcohol use in the previous week. Random effect predictors were participant identifier, module topics, and the interaction between module topics and the indicator of a high-risk drinking day. Similarly, favorability ratings were modelled with linear regression that included the following fixed effect predictors: age, sex, ASI alcohol composite score, clinical trial identifier, and number of days since treatment randomization. Random effect predictors were participant identifier, module topics, feedback form items, and the interaction between module topics and feedback form items.

Several variables were included as covariates in the statistical models to account for potential confounding. Two covariates were rescaled to improve MCMC convergence. Age was rescaled so that 21 years old was set to 0 and each 1-unit increase represented an increase of 10 years. The number of days since treatment randomization was rescaled so that each 1-unit increase represented an increase of 28 days. Missing data in the outcome and the predictors were assumed to be missing at random and were modelled jointly during the MCMC procedures^69^. Missing ASI alcohol composite scores were modelled with a zero-or-one inflated beta distribution because scores can range from 0 to 1^70^. Missing numbers of days since treatment randomization were modelled with a half-normal distribution to only allow positive numbers. Missing indicators of alcohol use in the previous week were modelled with a Bernoulli distribution because the indicator is a binary variable. The exact parameters used to model missing data can be found in the statistical analysis code. Noninformative prior distributions were specified to have minimal influence on the results for the fixed effects (*normal*(0, 1E6)) and residual variances (*igamma*(0.01, 0.01))^68^. Shrinkage priors were specified for the variances of the random effects (*half-t*(0, 10, 3)) to mitigate the inflation of false positives that can occur due to testing several hypotheses (“multiplicity”)^71,72^. None of the distributions assigned greater prior probability of superiority to any of the modules.

As a sensitivity analysis, we reanalyzed the data while only including data from participants who completed all 6 core modules. All MMRMs were conducted using PROC MCMC in SAS 9.4 by running 4 separate MCMC chains with different initial values^68^. Convergence of the MCMC chains was determined when parameters had $\hat{r}$ < 1.01 and effective sample sizes > 400 for the tail quantiles (5%, 95%) and the rank-normalized values^73^. The *posterior* package (v1.5.0) in R Statistical Software version 4.2.1 was used to generate $\hat{r}$ values and effective sample sizes^74,75^. The data and code are available at https://osf.io/encv6/ and generally follow the Clinical Data Interchange Standards Consortium Analysis Data Model (v1.3)^76^.

## Supplementary information

Supplementary information

## Data Availability

The deidentified data used for this study are publicly available at https://osf.io/encv6/.

## Code availability

The statistical code used for this study is publicly available at https://osf.io/encv6/.
