# Supplementary material for: Comparing content within a culturally-adapted digital treatment for Hispanic patients with alcohol use disorder
Source: NPJ Digit Med. 2025 Dec 8;9:23. doi: 10.1038/s41746-025-02197-7 (PMC12783792; doi:10.1038/s41746-025-02197-7)
Supplement: Supplementary file 1 — Supplementary information [file 41746_2025_2197_MOESM1_ESM.pdf]

Supplementary Information

The following example illustrates how missing data observations were structured in the alcohol use calendar dataset. Imagine that exactly 5 days after a participant completes a module (e.g., functional analysis), the participant completes the next module. Of the 5 days that occurred between the two modules, 4 were low-risk drinking days (Monday-Thursday) and 1 was a high-risk drinking day (Friday). The participant reported not drinking any alcohol on all 5 days, and the data is recorded on the participant's daily alcohol use calendar. See table below for an example of the longitudinal data structure.

Supplementary Table 1.

Participant ID	Module	Risk	Calendar Days	Alcohol Use Days
37022	functional analysis	low-risk	4	0
37022	functional analysis	high-risk	1	0

Because our longitudinal data structure requires that each module be linked to exactly 7 calendar days, 2 missing data points are inserted into the dataset. We do not know on how many of these missing days the participant would have used alcohol. We also do not know whether these days would have been low-risk or high-risk drinking days. See table below for the dataset that now includes missing data.

Supplementary Table 2.

Participant ID	Module	Risk	Calendar Days	Alcohol Use Days
37022	functional analysis	low-risk	4	0
37022	functional analysis	high-risk	1	0
37022	functional analysis	?	2	?

But because each calendar week must include at least 3 high-risk drinking days (Fridays-Sundays), the missing data are updated to reflect this fact. Therefore, the 2 missing daily data points are assumed to be high-risk drinking days. Now the functional analysis module is linked to 7 days in total; 4 calendar days are low-risk drinking days and 3 calendar days are high-risk drinking days. The number of missing days on which the participant would have used alcohol is still unknown. See table below for the dataset that contains the updated information.

Supplementary Table 3.

Participant ID	Module	Risk	Calendar Days	Alcohol Use Days
37022	functional analysis	low-risk	4	0
37022	functional analysis	high-risk	1	0
37022	functional analysis	high-risk	2	?

This longitudinal data structure is then analyzed with the Bayesian statistical models. The missing data are modelled during the Markov chain Monte Carlo (MCMC)

procedures, which creates samples from the probability distributions of the Bayesian models. Any other covariates with missing data are also modelled jointly during the MCMC procedures (see SAS code at https://osf.io/encv6/).

The following example illustrates how contrasts between the marginal means were generated from the statistical models. First, one marginal mean is created for each module ($\hat{\mu}_a$) based on the regression coefficients. Second, for each module, the average of the marginal means for the other five modules is created ($\hat{\mu}_g$). Third, the contrast is created by subtracting $\hat{\mu}_a$ from $\hat{\mu}_g$. See example table below (not real data).

Supplementary Table 4.

**Module**	$\hat{\mu}_a$	$\hat{\mu}_g$	**Contrast ($\hat{\mu}_a$ - $\hat{\mu}_g$)**
Functional analysis	1	3	-2
Assertive communication	4	2.4	1.6
Coping with craving	2.5	2.7	-0.2
Cognitive restructuring	3.5	2.5	1
Problem solving	3	2.6	0.4
Decision making	2	2.8	-0.8

In the example table above, the marginal mean for the functional analysis module is 1 ($\hat{\mu}_a = 1$). The average of the marginal means for the other five modules is 3 ($\hat{\mu}_g = [4 + 2.5 + 3.5 + 3 + 2] / 5 = 3$). The contrast between functional analysis and the other five modules is -2 ($\hat{\mu}_a - \hat{\mu}_g = 1 – 3 = -2$).

The process illustrated above was used to generate the results for both the logistic MMRM and the linear MMRM reported in the main text. The contrasts between $\hat{\mu}_a$ and $\hat{\mu}_g$ are the basis for the odds ratios (*OR*) and unstandardized differences (*b*) reported in *Table 3* and *Table 4* of the main text.

The results reported below are from the sensitivity analyses that included only participants who completed all 6 CBT4CBT-S modules. The table below shows contrasts in alcohol use between the CBT4CBT-S modules by daily risk of drinking (compare to *Table 3* in the main text).

Supplementary Table 5.

**Daily risk of drinking**	**Module**	***OR* [*95 CI*]**	***p*(*H_1*|*data*)**
Low-risk day	Functional analysis	2.45 [0.75, 6.11]	8%
	Assertive communication	1.36 [0.51, 3.07]	33%
	Coping with craving	0.68 [0.16, 1.68]	84%
	Cognitive restructuring	0.65 [0.14, 1.55]	87%
	Problem solving	0.66 [0.21, 1.45]	87%
	Decision making	2.29 [0.87, 5.26]	5%
High-risk day	Functional analysis	2.81 [1.05, 6.35]	2%
	Assertive communication	1.11 [0.49, 2.11]	45%
	Coping with craving	0.68 [0.26, 1.38]	87%
	Cognitive restructuring	0.71 [0.27, 1.42]	84%
	Problem solving	0.85 [0.36, 1.70]	73%
	Decision making	1.28 [0.57, 2.40]	31%

Note. Results for the Bayesian MMRM logistic regression model of daily alcohol use. Low-risk day = any day that was not a high-risk drinking day. High-risk day = Friday, Saturday, Sunday, or holiday (and the day before the holiday) associated with increased drinking. Odds ratios and posterior probabilities were based on contrasts between the marginal mean for each module ($\hat{\mu}_a$) and the average of the marginal means for the other modules ($\hat{\mu}_g$). *OR* = odds ratio. *95 CI* = 95% credibility interval. *p*(*H_1*|*data*) = posterior probability that the module is associated with less drinking than the other modules (H_1: $\hat{\mu}_a < \hat{\mu}_g$).

The table below shows contrasts in patient favorability ratings between CBT4CBT-S modules by feedback form item (compare to *Table 4* in the main text).

Supplementary Table 6.

**Item**	**Module**	***b* [*95 CI*]**	***p*(*H_1*|*data*)**
Effectiveness	Functional analysis	-0.04 [-0.25, 0.17]	37%
	Assertive communication	-0.08 [-0.27, 0.10]	19%
	Coping with craving	-0.05 [-0.25, 0.13]	28%
	Cognitive restructuring	0.16 [-0.02, 0.36]	96%
	Problem solving	-0.11 [-0.31, 0.07]	12%
	Decision making	0.13 [-0.07, 0.33]	90%
Novelty	Functional analysis	-0.04 [-0.26, 0.17]	34%
	Assertive communication	-0.09 [-0.28, 0.09]	16%
	Coping with craving	-0.03 [-0.21, 0.16]	37%
	Cognitive restructuring	0.12 [-0.09, 0.30]	88%
	Problem solving	-0.10 [-0.31, 0.09]	14%
	Decision making	0.15 [-0.04, 0.37]	94%
Applicability	Functional analysis	-0.04 [-0.25, 0.15]	35%

	Assertive communication	-0.07 [-0.25, 0.12]	22%
	Coping with craving	-0.05 [-0.24, 0.14]	31%
	Cognitive restructuring	0.14 [-0.03, 0.33]	94%
	Problem solving	-0.11 [-0.31, 0.08]	12%
	Decision making	0.13 [-0.06, 0.33]	90%
Navigation	Functional analysis	-0.03 [-0.24, 0.18]	39%
	Assertive communication	-0.09 [-0.29, 0.09]	16%
	Coping with craving	-0.03 [-0.22, 0.15]	36%
	Cognitive restructuring	0.15 [-0.02, 0.35]	95%
	Problem solving	-0.12 [-0.32, 0.07]	12%
	Decision making	0.12 [-0.07, 0.31]	89%
Enjoyability	Functional analysis	-0.03 [-0.24, 0.18]	40%
	Assertive communication	-0.10 [-0.29, 0.08]	15%
	Coping with craving	-0.06 [-0.25, 0.13]	26%
	Cognitive restructuring	0.15 [-0.03, 0.34]	95%
	Problem solving	-0.11 [-0.31, 0.08]	12%
	Decision making	0.14 [-0.04, 0.35]	93%
Relatability	Functional analysis	-0.02 [-0.21, 0.19]	43%
	Assertive communication	-0.11 [-0.30, 0.08]	12%
	Coping with craving	-0.03 [-0.22, 0.15]	35%
	Cognitive restructuring	0.14 [-0.05, 0.32]	92%
	Problem solving	-0.11 [-0.31, 0.08]	14%
	Decision making	0.13 [-0.07, 0.33]	91%

Note. Results for the Bayesian MMRM linear regression model of patient feedback form items. Differences and posterior probabilities were based on contrasts between the marginal mean for each module ($\hat{\mu}_a$) and the average of the marginal means for the other modules ($\hat{\mu}_g$). *b* = unstandardized mean difference. *95% CI* = 95% credibility interval. $p(H_1|data)$ = posterior probability of the hypothesis that the module was rated better than the other modules (H_1: $\hat{\mu}_a > \hat{\mu}_g$).

Supplementary Figure 1. CONSORT diagram for data from both clinical trials.

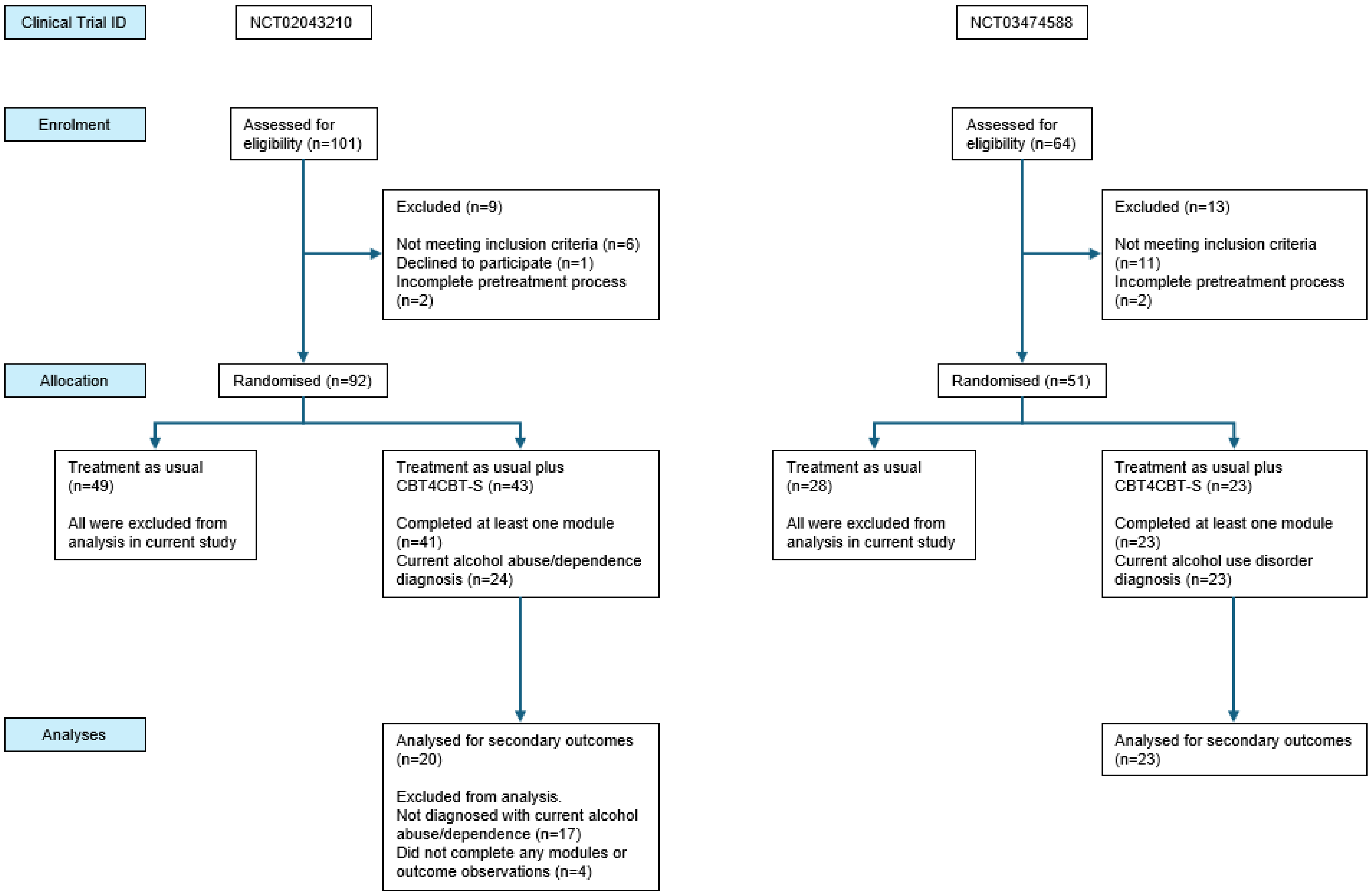

Supplementary Table 7. CONSORT checklist.

Section/topic	No	CONSORT 2025 checklist item description	Reported on page no.
**Title and abstract**			
Title and structured abstract	1a	Identification as a randomised trial	2
	1b	Structured summary of the trial design, methods, results, and conclusions	2
**Open science**			
Trial registration	2	Name of trial registry, identifying number (with URL) and date of registration	2, (References #31 and #42)
Protocol and statistical analysis plan	3	Where the trial protocol and statistical analysis plan can be accessed	2, (References #31 and #42)
Data sharing	4	Where and how the individual de-identified participant data (including data dictionary), statistical code and any other materials can be accessed	12
Funding and conflicts of interest	5a	Sources of funding and other support (eg, supply of drugs), and role of funders in the design, conduct, analysis and reporting of the trial	13
	5b	Financial and other conflicts of interest of the manuscript authors	13
**Introduction**			
Background and rationale	6	Scientific background and rationale	3-4
Objectives	7	Specific objectives related to benefits and harms	3-4
**Methods**			
Patient and public involvement	8	Details of patient or public involvement in the design, conduct and reporting of the trial	8-9
Trial design	9	Description of trial design including type of trial (eg, parallel group, crossover), allocation ratio, and framework (eg, superiority, equivalence, non-inferiority, exploratory)	8, 10
Changes to trial protocol	10	Important changes to the trial after it commenced including any outcomes or analyses that were not prespecified, with reason	8
Trial setting	11	Settings (eg, community, hospital) and locations (eg, countries, sites) where the trial was conducted	8
Eligibility criteria	12a	Eligibility criteria for participants	8
	12b	If applicable, eligibility criteria for sites and for individuals delivering the interventions (eg, surgeons, physiotherapists)	Not applicable
Intervention and comparator	13	Intervention and comparator with sufficient details to allow replication. If relevant, where additional materials describing the intervention and comparator (eg, intervention manual) can be accessed	8-9
Outcomes	14	Prespecified primary and secondary outcomes, including the specific measurement variable (eg, systolic blood pressure), analysis metric (eg, change from baseline, final value, time to event), method of aggregation (eg, median, proportion), and time point for each outcome	9-12

Harms	15	How harms were defined and assessed (eg, systematically, non-systematically)	4
Sample size	16a	How sample size was determined, including all assumptions supporting the sample size calculation	10
	16b	Explanation of any interim analyses and stopping guidelines	10
Randomisation			
Sequence generation	17a	Who generated the random allocation sequence and the method used	10
	17b	Type of randomisation and details of any restriction (eg, stratification, blocking and block size)	10
Allocation concealment mechanism	18	Mechanism used to implement the random allocation sequence (eg, central computer/telephone; sequentially numbered, opaque, sealed containers), describing any steps to conceal the sequence until interventions were assigned	8
Implementation	19	Whether the personnel who enrolled and those who assigned participants to the interventions had access to the random allocation sequence	8
Blinding	20a	Who was blinded after assignment to interventions (eg, participants, care providers, outcome assessors, data analysts)	8
	20b	If blinded, how blinding was achieved and description of the similarity of interventions	8
Statistical methods	21a	Statistical methods used to compare groups for primary and secondary outcomes, including harms	10-12
	21b	Definition of who is included in each analysis (eg, all randomised participants), and in which group	8
	21c	How missing data were handled in the analysis	11-12
	21d	Methods for any additional analyses (eg, subgroup and sensitivity analyses), distinguishing prespecified from post hoc	12
**Results**			
Participant flow, including flow diagram	22a	For each group, the numbers of participants who were randomly assigned, received intended intervention, and were analysed for the primary outcome	4
	22b	For each group, losses and exclusions after randomisation, together with reasons	4
Recruitment	23a	Dates defining the periods of recruitment and follow-up for outcomes of benefits and harms	4
	23b	If relevant, why the trial ended or was stopped	Not applicable
Intervention and comparator delivery	24a	Intervention and comparator as they were actually administered (eg, where appropriate, who delivered the intervention/comparator, how participants adhered, whether they were delivered as intended (fidelity))	4, 8-9
	24b	Concomitant care received during the trial for each group	8
Baseline data	25	A table showing baseline demographic and clinical characteristics for each group	4, (References #31 and #42)

Numbers analysed, outcomes and estimation	26	For each primary and secondary outcome, by group:<br>● the number of participants included in the analysis<br>● the number of participants with available data at the outcome time point<br>● result for each group, and the estimated effect size and its precision (such as 95% confidence interval)<br>● for binary outcomes, presentation of both absolute and relative effect size	4-5, Tables 1-4, Figure 1
Harms	27	All harms or unintended events in each group	4
Ancillary analyses	28	Any other analyses performed, including subgroup and sensitivity analyses, distinguishing pre-specified from post hoc	5
**Discussion**			
Interpretation	29	Interpretation consistent with results, balancing benefits and harms, and considering other relevant evidence	5-7
Limitations	30	Trial limitations, addressing sources of potential bias, imprecision, generalisability, and, if relevant, multiplicity of analyses	7
